# Our experience with enzyme-linked immunospot assay in the laboratory diagnostics of lyme borreliosis

**DOI:** 10.1186/1939-4551-8-S1-A121

**Published:** 2015-04-08

**Authors:** Andrea Ondrejkova, Iveta Spackova, Hana Svandova, Jana Chlebkova, Gustav Ondrejka

**Affiliations:** 1 Agel Laboratories, Czech Republic; 2 Hospital Novy Jicin, Czech Republic

## Background

Lyme borreliosis (LB) is multisystem infectious disease with various clinical manifestations. In Europe, LB is caused by gram-negative spirochetal bacteria of the complex *Borrelia burgdorferi* sensu lato. The most important for human pathogenicity are *B.garinii*, *B.afzelii* and *B.burgdorferi* sensu stricto. Indirect laboratory diagnostics of LB is based on serological methods such as Enzyme-Linked ImmunoSorbent Assay (ELISA) and Western Blot Assay (WB) that detect specific antibodies. Another method in the diagnostics of LB is a new test LymeSpot. This test is based on Enzyme-Linked ImmunoSpot Assay (ELISPOT). We present our first experience with the test LymeSpot.

## Methods

From November 2013 to June 2014 we performed LymeSpot on the group of 42 patients who were tested for suspicion of LB presence. LymeSpot is based on specific cell immune response of T-lymphocytes after stimulation by Borrelia antigens. The result is a number of antigen specific effector T-cells producing IFN-γ in the form of spots. We compared the results of LymeSpot test with the level of specific IgM and IgG antibodies detected by ELISA and WB with using recombinant antigens *B. burgdorferi* sl.

## Results

From the number of 42 patients we detected positive LymeSpot test only in 4 of them (9,5%), a cutoff value in 3 patients (7,1%). From the group of 42 patients were simultaneously performed ELISA and WB in 26 persons. In this group we found positive LymeSpot in 4 patients and cutoff value in 2 patients. In the group of 4 patients with positive LymeSpot was detected higher level of IgM antibodies in 3 patients and only IgG antibodies in 1 patient. They were patients with clinical manifestations of LB who were treated with antibiotics afterwards. Among 20 patients with negative results of LymeSpot these findings correlated with antibody response in 8 patients (40%). In this group we detected seronegative finding (IgM-IgG-) or finding of passing through LB (IgM-IgG+). In other 12 patients with negative LymeSpot test we detected serological finding of beginning (IgM+IgG-) or running LB (IgM+IgG+). They were mostly patients without clear symptoms of LB, patients with long-term positivity of IgM antibodies without clinical manifestations of LB or patients after antibiotic therapy.

## Conclusions

Our first experience shows that results of LymeSpot test better correlate with clinical findings and LB activity as well as can be effective marker of success of antibiotic therapy.

